# Overexpressed CMTM6 Improves Prognosis and Associated With Immune Infiltrates of Ovarian Cancer

**DOI:** 10.3389/fmolb.2022.769032

**Published:** 2022-01-31

**Authors:** Bo Yin, Jianyi Ding, Haoran Hu, Meiqin Yang, Baoyou Huang, Wei Dong, Fang Li, Lingfei Han

**Affiliations:** ^1^ Department of Gynecology, Shanghai First Maternity and Infant Hospital, School of Medicine, Tongji University, Shanghai, China; ^2^ Department of Gynecology, Shanghai East Hospital, School of Medicine, Tongji University, Shanghai, China

**Keywords:** CMTM6, prognosis, biomarker, immunity, ovarian cancer

## Abstract

Ovarian cancer (OV) is an epithelial malignancy that intrigues people for its high mortality and lack of efficient treatment. Chemokine-like factor (CKLF)–like MARVEL transmembrane domain containing 6 (CMTM6) can be observed in various cancers, but its part in OV remains little known. Hence, the prognostic value and underlying mechanism of CMTM6 in OV were preliminarily evaluated. Here, we determined that CMTM6 expression was higher than that in normal controls. However, the upregulation of CMTM6 was associated with better prognosis. GSEA results suggested that CMTM6 is involved in the immune-related and metabolism-related pathways. GO/KEGG analysis of CMTM6 coexpressed genes was performed to survey the possible regulatory roles of CMTM6 in OV. Subsequently, CMTM6 expression was positively correlated with the infiltration levels of immune cells and the expression of diverse immune cell marker sets. Importantly, CMTM6 may influence prognosis partially by regulating immune infiltration in OV. Last, copy number variations (CNVs) and DNA methylation might prompt the abnormal CMTM6 expression in OV. In conclusion, CMTM6 can serve as a novel prognostic biomarker in patients with OV.

## Introduction

The fatality rate of ovarian cancer (OV) is the highest among all female reproductive tract tumors (Ferlay et al., 2015). Most of the cases are diagnosed at an advanced stage, which results in poor prognosis of this disease. Although breakthrough has been made in the screening and prevention and targeted therapies to improve the survival of OV patients, there was no definitive mortality reduction (Lisio et al., 2019). In view of these circumstances, it is imperious to discover a promising biomarker or new targets that make substantial contribution to improve patient prognosis.

The human chemokine-like factor (CKLF)–like MARVEL transmembrane domain-containing (CMTM) is a gene family consisting of nine members, CKLF and CMTM1–8 (Han et al., 2001; Han et al., 2003). Their encoded products are structurally and functionally intermediate classical chemokines and the transmembrane 4 superfamily (TM4SF), which play important roles in the immune system, tumorigenesis, cell cycle, and the male reproductive system (Zheng et al., 2011; Li et al., 2014; Liu et al., 2019).

CMTM6 is located in the p22 region of chromosome 3; like other members of this family, the CMTM6 protein is a type 3 transmembrane protein with MARVEL-like domains containing at least three transmembrane spiral structures. In addition to its predicted localization at the plasma membrane, CMTM6 is also located in the cytoplasm or on the intermediate filament (Mamessier et al., 2018). CMTM6 has been described as a programmed death ligand 1 (PD-L1) regulator at the protein level *via* modulating stability through ubiquitination (Burr et al., 2017; Mezzadra et al., 2017). Other studies have pointed out that the deletion of CMTM6 can reduce tumor-specific T cell activity and cause tumor “immune escape,” thus showing that CMTM6 may be a tumor suppressor gene in tumorigenesis (Zhu et al., 2019), but at the same time, more studies have indicated that CMTM6 is highly expressed in head and neck squamous cell carcinoma, hepatocellular carcinoma, non–small cell lung cancer, and gliomas and showed a poor prognosis (Guan et al., 2018; Lisio et al., 2019; Chen et al., 2020; Liu et al., 2021). This suggests that CMTM6 may have different regulatory effects in different tumors. Nevertheless, little is known about the specific role and prognostic value of CMTM6 in OV.

In this study, we first performed a series of bioinformatics analyses on CMTM6 in OV consisting of transcriptional analysis, coexpression analysis, functional annotation enrichment analysis, protein–protein interaction (PPI) analysis, and survival analysis. Besides, further exploration was carried out to estimate the status of CMTM6 in the tumor microenvironment using TIMER 2.0, TISIDB, and GEPIA2 databases. The epigenetic alteration of CMTM6 is connected with its dysregulation. Collectively, the purpose of this article is to ascertain if CMTM6 could be a new potential prognostic biomarker for OV and hopefully to help filter OV patient’s suitable prognostic indicators for the therapy method.

## Materials and Methods

### Analysis of CMTM6 Gene Expression in Multiple Platforms

A pan-cancer analysis of CMTM6 was conducted by the GEPIA2 (Tang et al., 2019). The CMTM6 mRNA expression level was determined in OV and their corresponding normal tissues in Oncomine, and the cutoff of p value and fold change was as following: p value:0.05; fold change:2; and gene rank:10% (Rhodes et al., 2007). Besides, we investigated the CMTM6 expression level based on The Cancer Genome Atlas (TCGA) database and the Genotype-Tissue Expression (GTEx) database in 427 tumor samples and 88 normal tissues by R package “ggplot2” visualization and log2 (TPM+1) for log-scale. CMTM6 expression in 55 OV cell lines was identified in the Cancer Cell Line Encyclopedia (CCLE) datasets (Barretina et al., 2012). Immunohistochemistry (IHC) images from the Human Protein Atlas (HPA) database were used to manifest the CMTM6 protein expressed level. Eventually, the UALCAN platform was queried to examine the correlation between CMTM6 gene expression and clinical features. These pre-defined subgroups include individual cancer stages, patient’s race, patient’s age, tumor grade, and TP53 mutation status. The difference was made by comparing median values.

### Cell Culture

Normal ovary cell line (IOSE80) and ovarian cancer cell lines (A2780, ES2, Hey, and SKOV3) obtained from the American Type Culture Collection (ATCC, Manassas, VA, United States) were cultured in Roswell Park Memorial Institute (RPMI)1,640 medium (HyClone, United States) supplemented with 10% fetal bovine serum and 1% penicillin/streptomycin. All cell lines were maintained in a humidified incubator in an atmosphere of 5% CO_2_ at 37°C.

### RNA Extraction, Reverse Transcription, and qRT-PCR

Total RNA of cell lines was extracted using RNAiso Plus reagent (TaKaRa, Shanghai, China) and transcribed into cDNA with the reverse transcription kit (TaKaRa) according to the manufacturer’s protocol. Quantitative real-time PCR was performed using cDNA primers specific for mRNA. All the real-time PCR reactions were performed using the QuantStudio^TM^ Design and Analysis Software1.3.1 PCR System. The 2^−ΔΔCt^ method was used for quantification, and fold change for targeted genes was normalized by internal control. The PCR reaction conditions were as follows: 95°C for 30 s, followed by 95°C for 5 s, and 60°C for 30 s: 40 cycles and then 95°C for 15 s, 60°C for 1 min, and 95°C for 15 s again. The expression levels were normalized against those of the internal reference gene GAPDH. The primer sequences are listed as follows: CMTM6 forward 5′-ATG​AAG​GCC​AGC​AGA​GAC​AG-3′, reverse 5′-GTG​TAC​AGC​CCC​ACT​ACG​GA-3′; GAPDH forward 5′-CGC​TCT​CTG​CTC​CTC​CTG​TTC-3′; reverse: 5′-ATC​CGT​TGA​CTC​CGA​CCT​TCA​C-3′.

### Western Blotting

The cells were lysed for 30 min with RIPA buffer and then centrifuged at 12,000 g for 30 min at 4°C to collect the supernatants. After mixing with SDS sample buffer, the proteins were heated to 99°C for 10 min, separated on 12.5% SDS-polyacrylamide gels, transferred to PVDF membranes, and probed with antibodies against CMTM6 (1:1,000, CST, United States) and actin (1:1,000, CST, United States) at 4°C overnight. A 1:2000 dilution of the HRP-linked anti-IgG (CST, United States) was used as secondary antibody, and blots were visualized by ECL (Millipore, United States), photographed using a cooled Tanon chemiluminescence gel imaging system (Thermo Fisher, United States), and band intensities were quantified using ImageJ software. The results were expressed as a ratio of band density to actin.

### Survival Analysis and Prediction

To uncover the prognostic value of the CMTM6 gene in OV patients, survival analysis, for instance overall survival (OS) and disease-free survival (DFS), was conducted. Relevant data of OV patients were downloaded from TCGA datasets, which were visualized with Kaplan–Meier curves. The analyses were accomplished through the R packages “survival” and “survminer” (Liu et al., 2018). The p-value was calculated by the log-rank test. A statistically significant variation was observed when the p-value was <0.05. The time-dependent receiver operating characteristic (ROC) curve was used to judge the predictive accuracy of the prognostic significance by using the pROC package (Vivian et al., 2017).

The Kaplan–Meier plotter database was further wielded to estimate the prognostic value between gene expression and survival in cancers (Lanczky et al., 2016). The relations between CMTM6 expression and survival in OV were validated by the PrognoScan database (Mizuno et al., 2009). Log-rank p-values and the hazard ratio (HR) with 95% confidence intervals (95% CIs) were calculated.

In some situations, GEPIA2 was also used to evaluate the prognostic value of CMTM6.

### Gene Set Enrichment Analysis

GSEA is a powerful analytical method to predict whether a gene set is enriched in a specific biological state. The clusterProfiler package in R was used for GSEA to reveal the critical biological process and relevant signaling pathways affected by CMTM6 expression. The cutoff criteria were *p* < 0.05 and normalized enrichment score (NES) > 1.

### Coexpressed Genes and PPI Network Analysis

The top 100 coexpressed genes with CMTM6 were extracted from coexpression analysis datasets in the Oncomine database. Fold change≥2 and P-value≤0.001 were selected as thresholds.

The GeneMANIA database is a resource-rich website containing gene information, analyzes gene lists, and prioritizes genes for functional assays with a high accuracy of the prediction algorithm (Warde-Farley et al., 2010). A figure where nodes symbolize genes and links represent protein–protein network (PPI) of coexpressed genes was used to display interactions. Cytoscape (version 3.8.2), an open bioinformatics software platform, was used to visualize the molecular interaction network.

### GO Term and KEGG Pathway Analysis

The Gene Ontology (GO) analysis comprising cellular component (CC), molecular function (MF), and biological process (BP), as well as the Kyoto Encyclopedia of Genes and Genomes (KEGG) pathway analysis of coexpressed genes were implemented *via* the R package clusterProfiler (3.14.3) (Yu et al., 2012). *P* < 0.05 was considered statistically significant for functional enrichment analysis.

### Systematic Analysis of Immune Characteristics

TIMER2.0 (tumor immune estimation resource, version 2) is a comprehensive resource for systematical analysis of immune cell infiltrate across diverse cancer types (Li et al., 2020). Spearman’s analysis was executed to illuminate the correlation between CMTM6 expression and the degree of immune infiltrates by the “Gene” module, and log2 (TPM) was used for log-scale.

TISIDB, well known as a web portal for tumor and immune system interaction, integrates multiple heterogenous data types (Ru et al., 2019). Here, TISIDB was used to explore the links between CMTM6 and multiple immune regulatory factors.

Meanwhile, the relationships between the expression of CMTM6 and gene markers of immune cells by the “correlation analysis” module of GEPIA2 were analyzed.

### Epigenetic Analysis

UCSC Xena is available to inspect gene expression, copy number, methylation, and somatic mutation in TCGA OV patient cohort (Goldman et al., 2020).

The genomic alteration types, alteration frequency, and CMTM6 mRNA level vs. methylation in OV were analyzed through the “OncoPrint” and “Plots” module, and the survival situation of the altered group or not were analyzed by the “Comparison/Survival” module in cBioPortal (Gao et al., 2013).

### Statistical Analysis

Partial statistical analyses were performed by default by website recourse. The Wilcoxon rank sum test was adopted to compare the CMTM6 expression levels in tumor tissues and normal tissues based on TCGA and GTEx databases. All R packages were chosen by R software version 3.6.3. The box plots were generated with GraphPad Prism 9 software, data are presented as mean ± standard deviation, and one-way analysis of variance (ANOVA) was used to determine the differences of CMTM6 expression in cell lines. All cell experiments had been repeated at least three times. Only P value < 0.05, adjusted *p* < 0.05, and log-rank p value < 0.05 were thought to be statistical significant (*p < 0.05; **p < 0.01; ***p < 0.001; ****p < 0.0001).

## Results

### The Expression of CMTM6 in Pan-Cancer and OV

**FIGURE 1:**
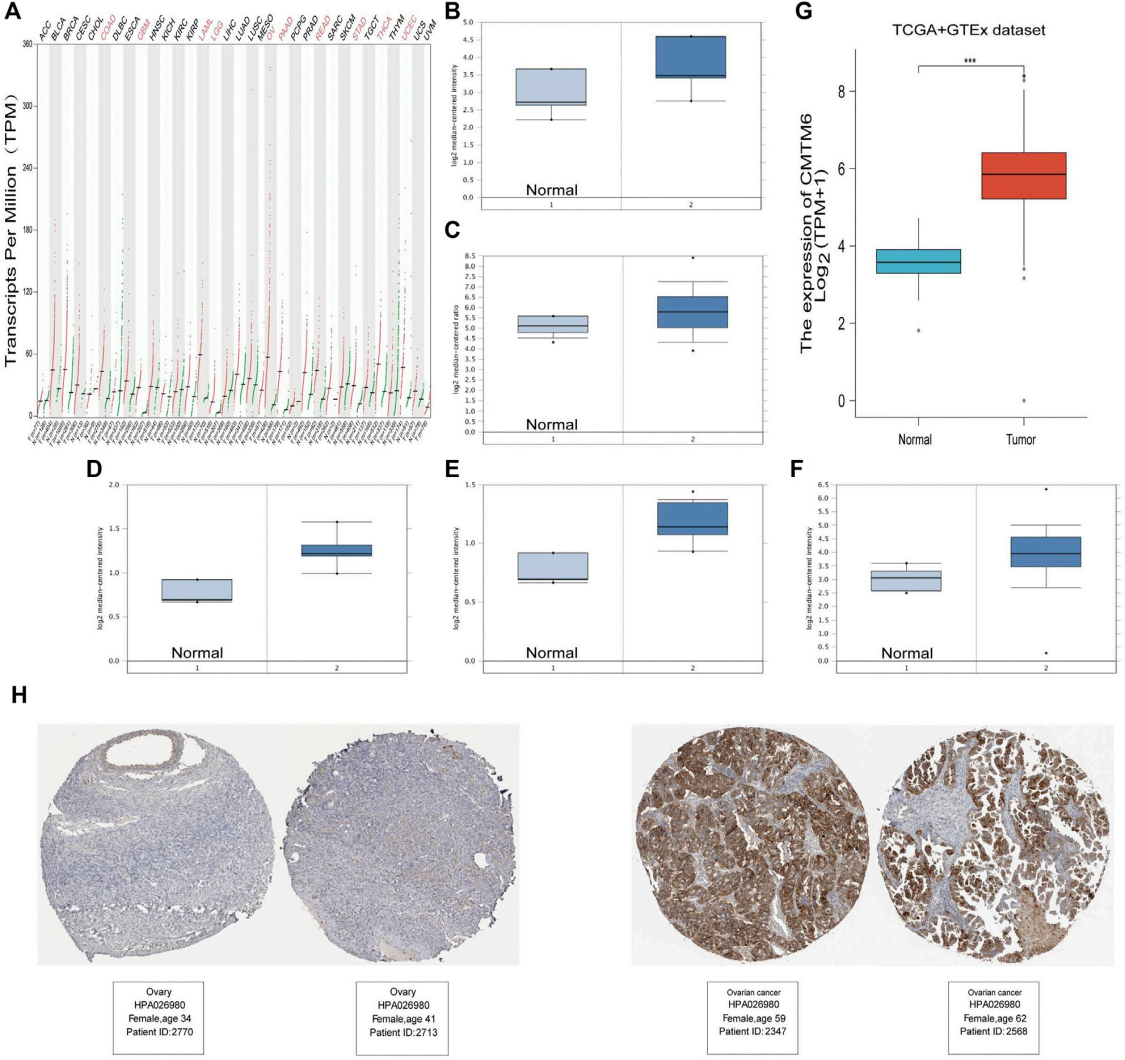
High expression of CMTM6 in various cancers and OV. **(A)** mRNA level of CMTM6 in human cancer tissues and corresponding normal tissues from GEPIA2. The expression of CMTM6 in the OV samples derived from the Oncomine database. Data shown for **(B)** ovarian endometrioid adenocarcinoma, **(C)** ovarian serous adenocarcinoma, **(D)** ovarian clear cell adenocarcinoma, **(E)** ovarian mucinous adenocarcinoma, and **(F)** ovarian carcinoma. **(G)** CMTM6 expression in OV matched TCGA and GTEx data. **(H)** Representative immunohistochemistry images and detailed information on the expression of CMTM6 in OV tissues and non-tumor tissues based on the THPA database. ***P < 0.001.

According to the GEPIA2 database query, we found that CMTM6 expression was upregulated in COAD (colon adenocarcinoma), GBM (glioblastoma multiforme), LAML (acute myeloid leukemia), LGG (brain lower grade glioma), OV (ovarian serous cystadenocarcinoma), PAAD (pancreatic adenocarcinoma), READ (rectum adenocarcinoma), STAD (stomach adenocarcinoma), THCA (thyroid carcinoma), and UCEC (uterine corpus endometrial carcinoma), especially in OV, compared with the corresponding normal tissues (Figure 1A). Consistently, higher mRNA expression of CMTM6 by the Oncomine database was observed in OV patients than that in normal samples (Figures 1B–F). The detailed results of CMTM6 expression in the Oncomine database are summarized in Table 1. Meanwhile, CMTM6 was also apparently increased when combining TCGA (427 tumor tissues) with GTEx (88 non-tumor tissues) (Figure 1G). In the end, representative IHC images from the THPA database further investigate that the CMTM6 protein level was obviously elevated in OV tissue compared with normal ovary tissue (Figure 1H). Therefore, we chose CMTM6 for further exploration.

**TABLE 1:** CMTM6 expression in diverse subtypes of OV and normal tissues using the Oncomine database.

Ovarian cancer subtype	Fold change	*t*-Test	P-value	Patient number	Reference
Ovarian endometrioid adenocarcinoma	1.806	2.653	0.012	14	PMID: 15161682
Ovarian serous adenocarcinoma	1.673	3.465	6.87E-04	53	PMID: 19486012
Ovarian clear cell adenocarcinoma	1.408	5.952	1.40E-04	12	PMID: 16452189
Ovarian mucinous adenocarcinoma	1.333	5.645	4.25E-04	17	PMID: 16452189
Ovarian carcinoma	1.888	6.779	1.48E-06	195	PMID: 18593951

Then, we dug the CMTM6 expression among groups of patients in line with different clinical parameters by using the UALCAN online tool, such as individual cancer stages, race, age, tumor grade, and TP53-mutation status. In terms of individual cancer stages, without increasing trend of CMTM6 level was witnessed as the aggravation of stages (Supplementary Figure S1A). Regrettably, regarding tumor grade, we could not know the effect of CMTM6 expression on tumor progression due to lack of clinical patient data for grade I and IV (Supplementary Figure S1B). Additionally, there were no differences in race, age groups, or TP53-mutation status (Supplementary Figures S1C–E).

### CMTM6 Expression in the OV Cell Lines

**FIGURE 2:**
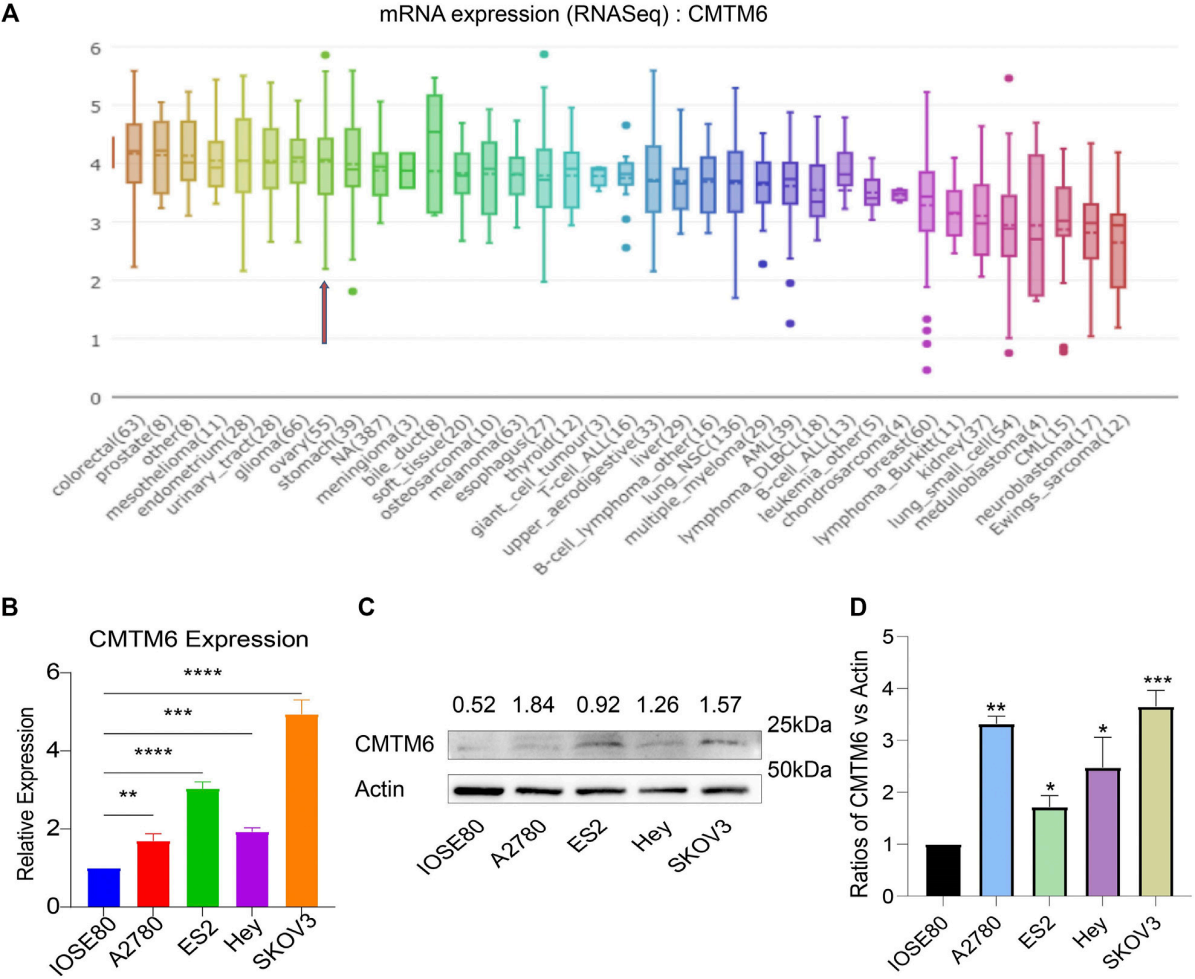
CMTM6 expression in OV cell lines. **(A)** Cell line indicated by the red arrow is ovarian cancer. CMTM6 was overexpressed in OV cell lines. **(B)** CMTM6 mRNA expression in human OV cell lines. **(C,D)** CMTM6 protein expression in human cell lines. Densitometric quantification was shown. Actin served as the loading control. *p < 0.05, **p < 0.01, ***p < 0.001, and ****p < 0.0001.

The data of cancer cell lines in the CCLE provided a large body of support about the expression of genes on many more cancer subtypes of various tissues of origin. With the use of the CCLE database, we discovered that CMTM6 was overexpressed in all cell lines of ovarian cancer (Figure 2A). Afterward, we examine the expression of CMTM6 mRNA and protein in four OV cell lines (A2780, ES2, Hey, and SKOV3) and human normal ovarian epithelial cell (IOSE80) by means of qRT-PCR and Western blot separately. The results confirmed that CMTM6 expression level was upregulated in OV cell lines compared to normal ovarian epithelial cells (Figures 2B–D). These results implied that high expression of CMTM6 may be closely correlated with the biological features of OV.

### Prognostic Value of CMTM6 Expression

**FIGURE 3:**
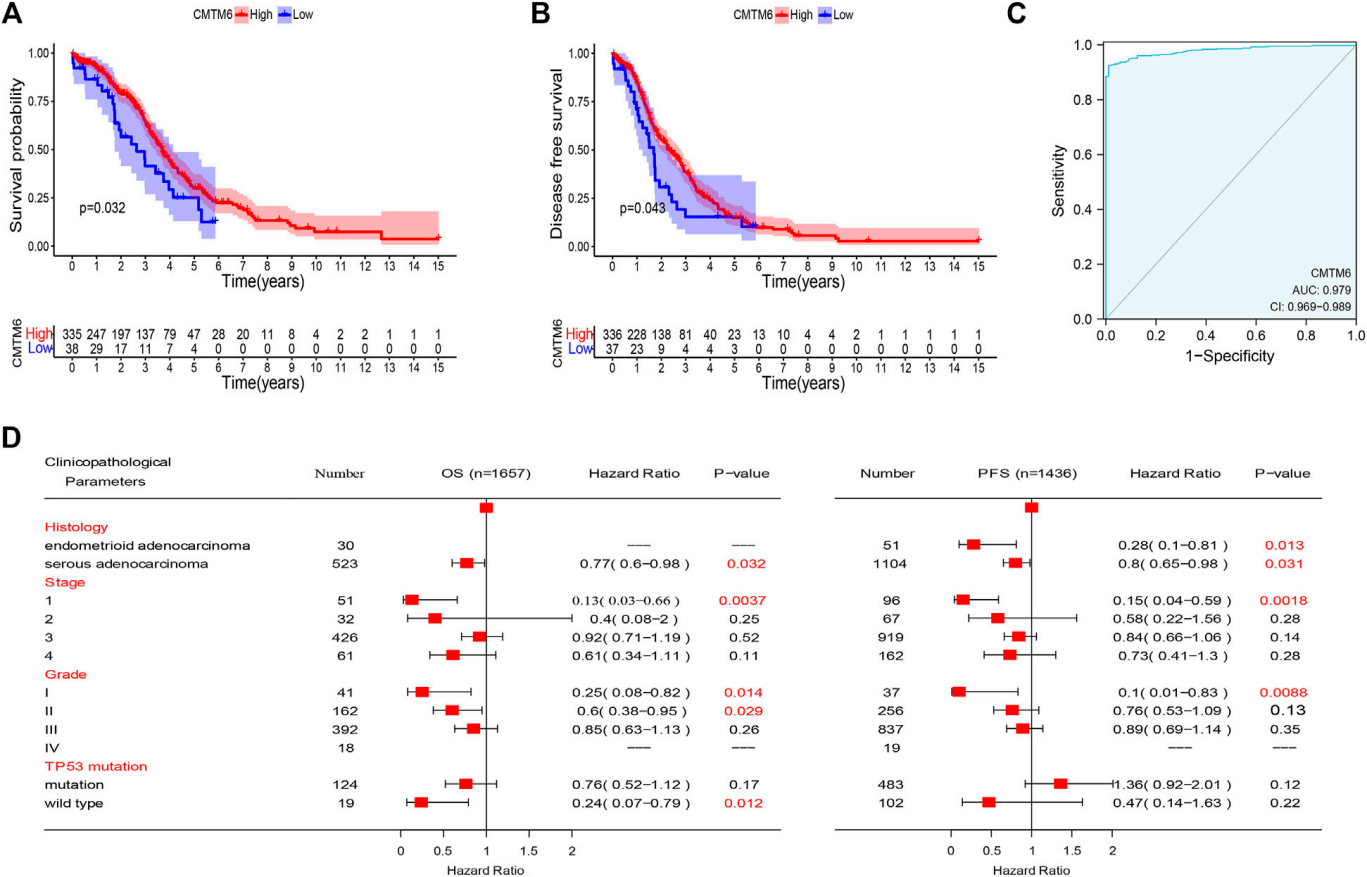
Prognostic value of CMTM6 expression and ROC analysis. **(A,B)** Survival analysis of CMTM6 in OV showed that high CMTM6 expression has an increased OS and DFS based on TCGA database. **(C)** ROC curve indicated the better performance of survival prediction. **(D)** Forest plot showed the correlation between CMTM6 expression and clinicopathological parameters in OV patients.

Based on the abovementioned consequences, we hypothesized that CMTM6 expression could well predict the survival of OV patients. To evaluate the role of CMTM6 in the OV patient survival, we classified the cancer cases from TCGA cohort into high-expression and low-expression groups. An unexpected observation in this regard is that the influence of CMTM6 overexpression in OV is related to preferable OS (Figure 3A, log-rank *p* = 0.032), and DFS (Figure 3B, log-rank *p* = 0.043), compared with low expression of CMTM6.

After that, we took advantage of the PrognoScan database to further verify that the elevated expression of CMTM6 has better prognosis in OV (Table 2). Although the outcomes in GSE17260 and GSE14764 were not statistically discrepant, we observed the similar tendency, which we attributed to insufficient samples. Moreover, ROC analysis was conducted on data from TCGA and GTEx to measure the discrimination value of CMTM6. The area under the curve (AUC) was 0.979 (Figure 3C). These outcomes not only strongly indicate a relevant role that high CMTM6 expression has with increased OS and DFS in OV but it is also a marker for poor prognosis in at least four solid tumors (Supplementary Figure S2). These puzzling findings caused us to further research the function of CMTM6 in OV.

**TABLE 2:** CMTM6 expression and survival data of OV by the PrognoScan database.

Dataset	End point	Probe ID	Number	Minimum P-value	HR
GSE9891	OS	223,047_at	278	0.000094	0.84 [0.65–1.08]
GSE26712	DFS	217,947 _at	185	0.000108	0.72 [0.61–0.85]
GSE26712	OS	217,947 _at	185	0.000710	0.72 [0.60–0.86]
GSE17260	OS	A_23_P57736	110	0.054256	0.85 [0.61–1.19]
GSE17260	DFS	A_23_P57736	110	0.059298	0.90 [0.69–1.18]
GSE14764	DFS	217,947_at	80	0.087340	0.68 [0.34–1.36]

### Check of the Prognostic Value of CMTM6 According to Diverse Clinicopathological Parameters

To better comprehend the prognostic role and assess the effect of CMTM6, we attempted to probe the connection between CMTM6 mRNA expression and clinical features applying the Kaplan–Meier database. We noticed that high CMTM6 expression in ovarian serous adenocarcinoma was linked to preferable OS and progression-free survival (PFS), but in ovarian endometrioid adenocarcinoma, high-expressed CMTM6 only exhibited decent OS (Figure 3D). Regarding different tumor stages, higher expression of CMTM6 only appeared beneficial to OS and PFS in the first stage of OV patients (Figure 3D). Similarly, a significant association between CMTM6 expression and superior OS in grade I and II was observed; however, better PFS can be found only in pathologic grade I (Figure 3D). Furthermore, CMTM6 upregulation was associated with positive OS in OV patients without TP53 mutation (Figure 3D). Hence, these analyses illustrate the prognostic value of the expression of CMTM6 for patients with OV.

### GSEA Enrichment Analysis Results of CMTM6

**FIGURE 4:**
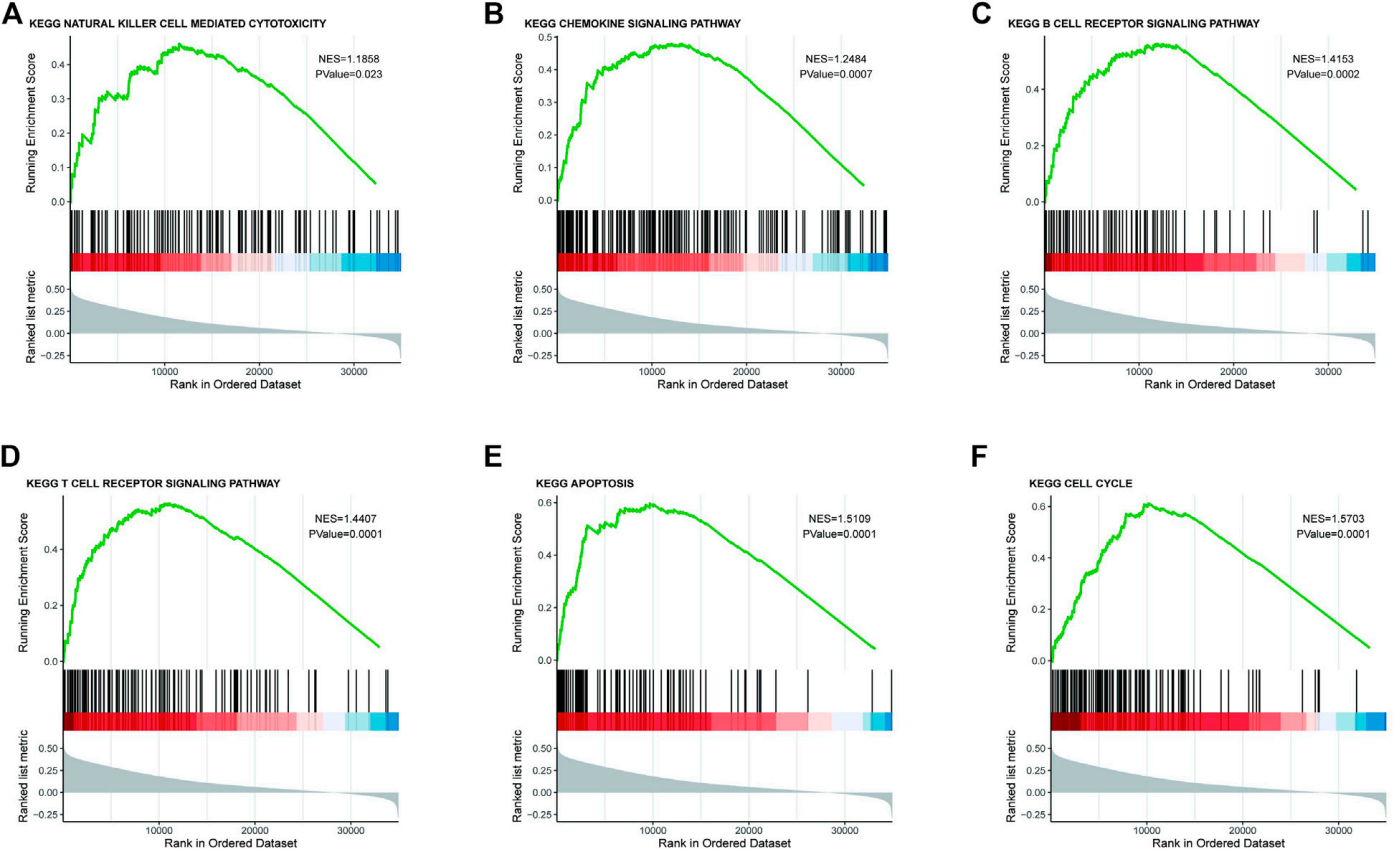
Enrichment plots from the GSEA. GSEA results showing **(A)** natural killer cell–mediated cytotoxicity, **(B)** chemokine signaling pathway, **(C)** B cell receptor signaling pathway, **(D)** T cell receptor signaling pathway, **(E)** apoptosis, and **(F)** cell cycle.

As CMTM6 expression is positively correlated with survival of OV, we tried to identify signaling pathways modulated by CMTM6 expression level. The GSEA was conducted, and biological pathways were enriched in the natural killer cell–mediated cytotoxicity, chemokine signaling pathway, B cell receptor signaling pathway, and T cell receptor signaling pathway (Figures 4A–D), which are immune-related pathways and in favor of antitumor effect. That might partially explain the connection of CMTM6 with beneficial prognosis; at the same time, it is found that GSEA results are also enriched in apoptosis (Figure 4E) and cell cycle (Figure 4F). Surprisingly, some metabolism pathways, such as adipocytokine signaling pathway, alanine aspartate and glutamate metabolism, fructose and mannose metabolism, and ubiquitin-mediated proteolysis were equally enriched (Supplementary Figure S3A). These certified that CMTM6 is involved in the immune response and metabolic control in OV.

### Coexpressed Genes of CMTM6 and Functional Enrichment Analysis

**FIGURE 5:**
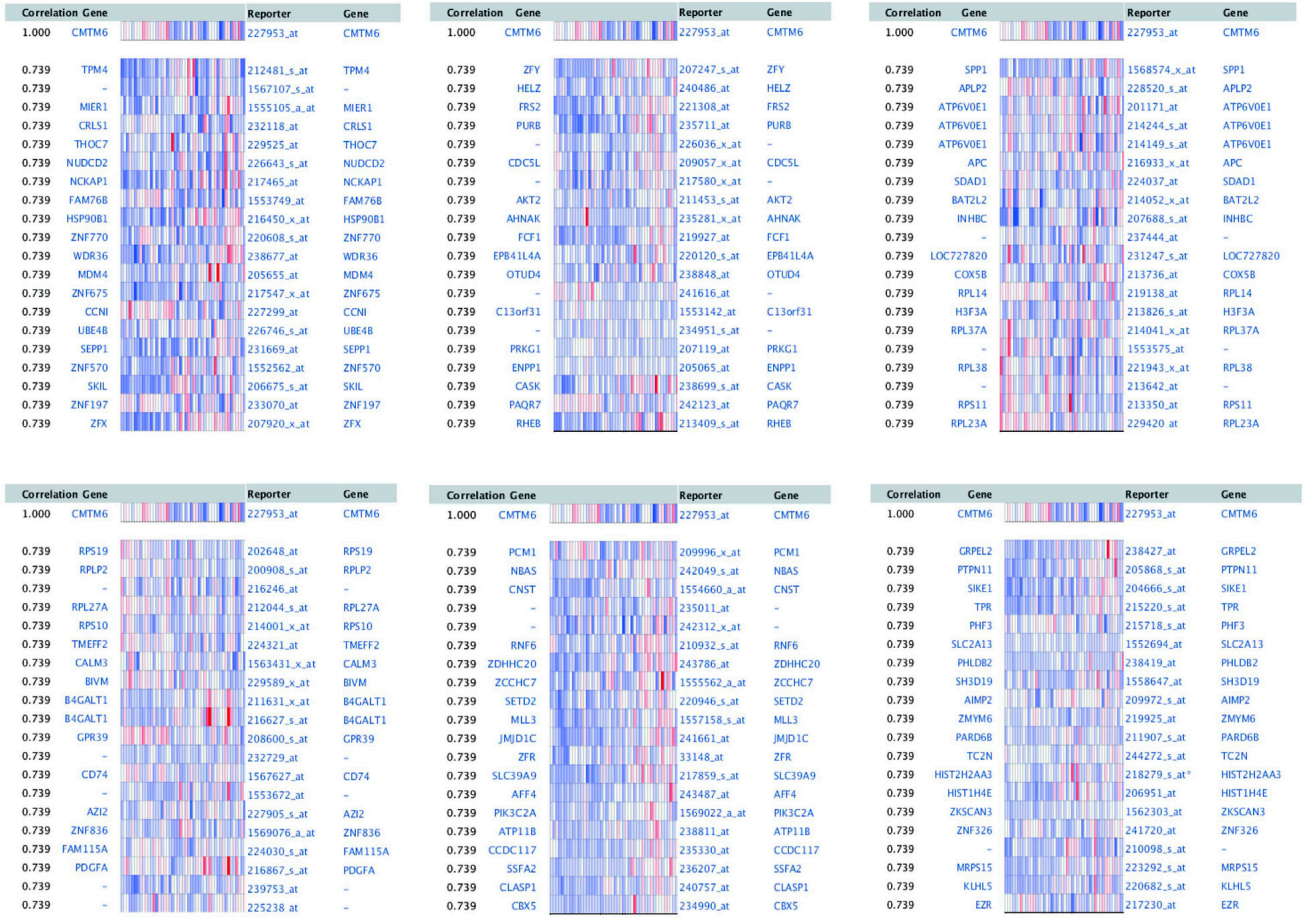
First 100 genes coexpressed with CMTM6 in OV were screened from Tothill Ovarian in the Oncomine database.

**FIGURE 6:**
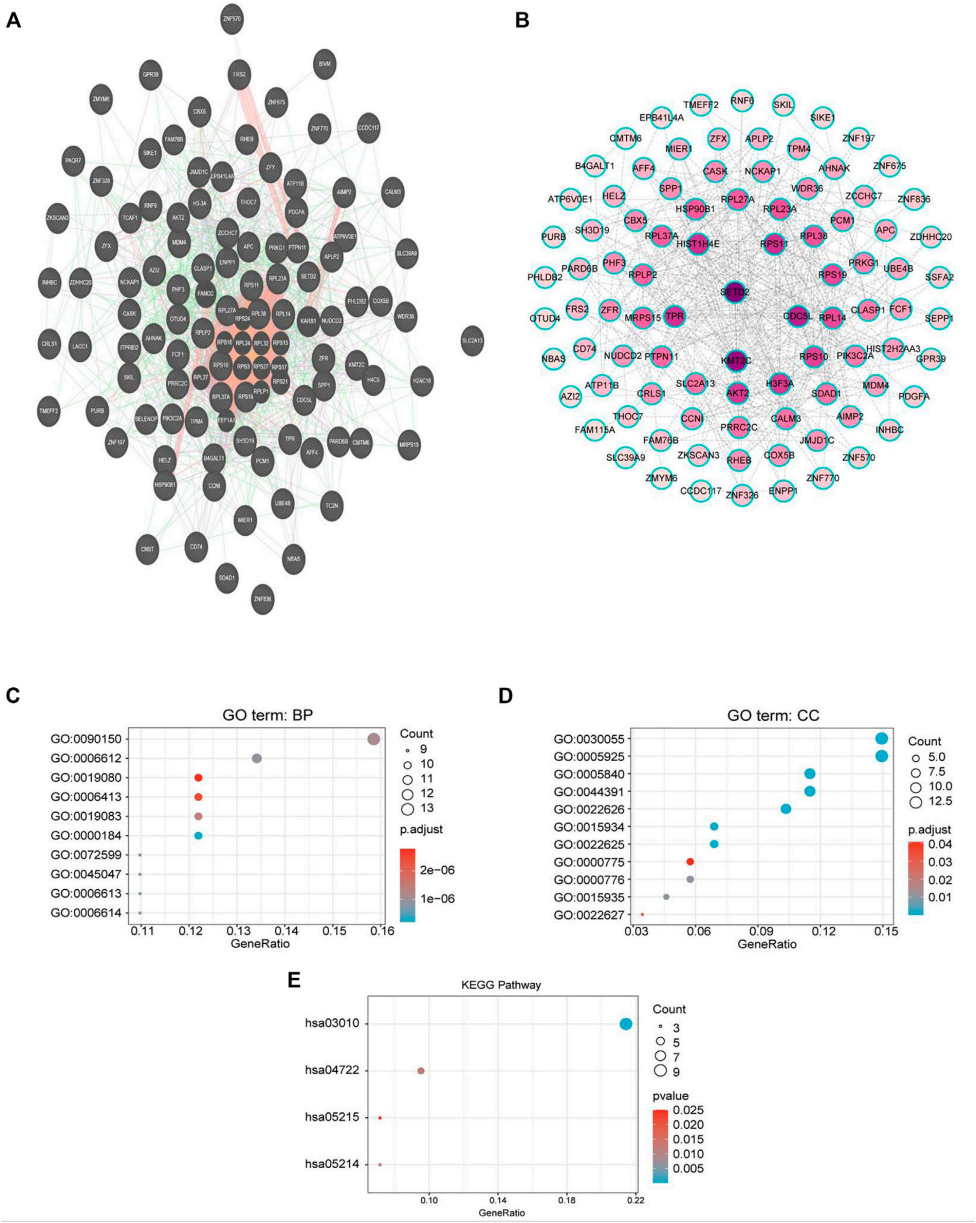
PPI network and GO/KEGG enrichment analysis of genes coexpressed with CMTM6. **(A)** PPI network of genes coexpressed with CMTM6 constructed in the GeneMANIA database. **(B)** Visualization of a GeneMANIA-derived network complex of molecular interactions in Cytoscape pathway visualization. **(C,D)** Enriched GO terms in the “biological process” and enriched GO terms in the “cellular component”; the top 10 are displayed. **(E)** KEGG pathway annotations. The X-axis represented the proportion of coexpressed genes, and the Y-axis represented different categories. The different colors indicate different properties, and the different sizes represent the number of genes.

**FIGURE 7:**
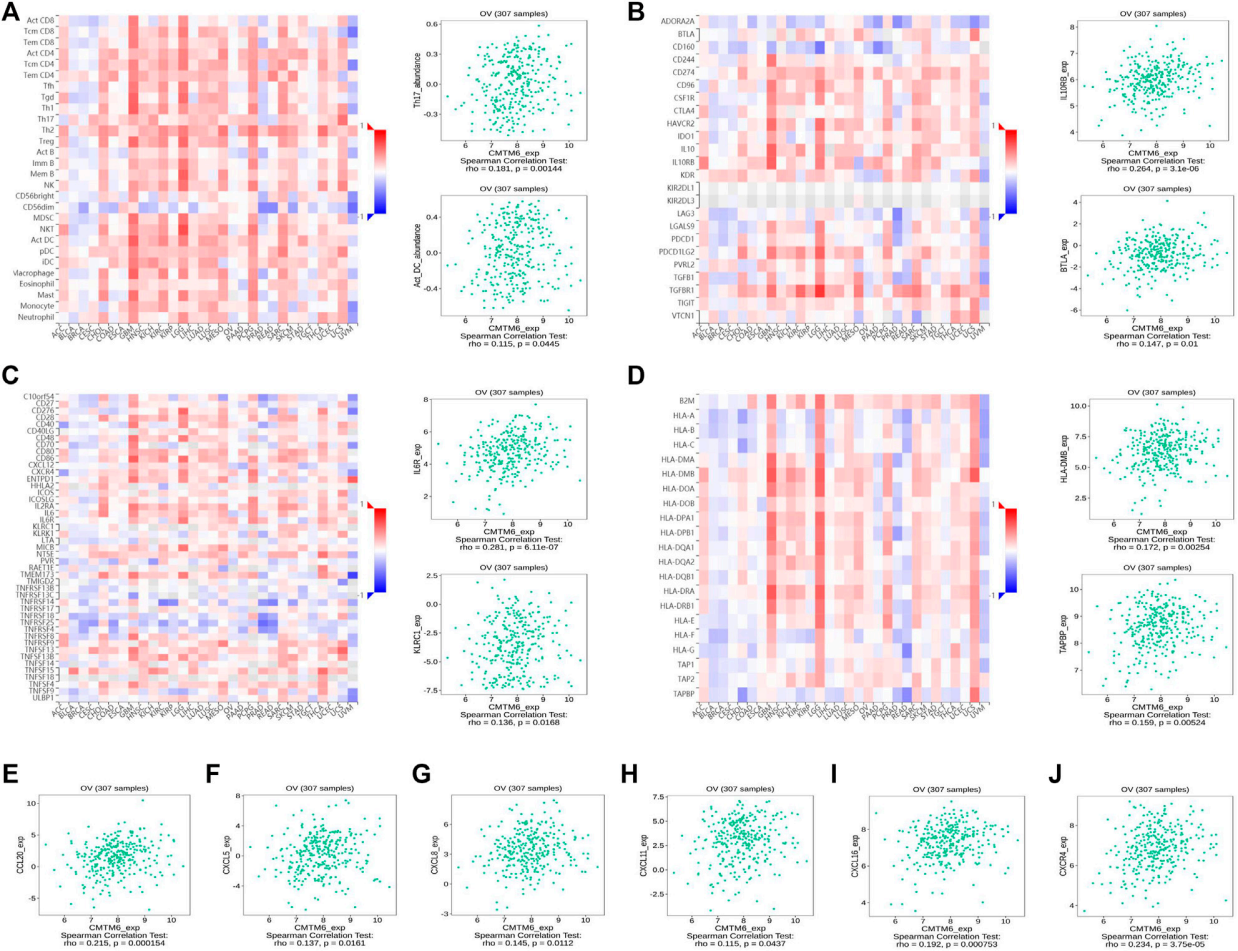
CMTM6 expression was associated with immune infiltration in OV from the TISIDB database. **(A–D)** Spearman relations between the abundances of TILs **(A)**, immunoinhibitors **(B)**, immunostimulators **(C)**, and MHC molecules **(D)**; the top 2 are exhibited. The relationship of CMTM6 expression with **(E–I)** chemokines and **(J)** chemokine receptor.

To explore the biological significance of CMTM6 in OV, we performed coexpression data mining to obtain the top 100 coexpressed genes of CMTM6 from a large cluster of 19,574 genes across Tothill Ovarian 295 samples in the Oncomine database (Figure 5). A PPI network analysis of coexpressing genes was generated by utilizing the GeneMANIA database (Figure 6A) and moved to the bioinformatics software platform Cytoscape (Version 3.8.2) to visualize the interaction network (Figure 6B). Afterward, to obtain a more extensive and in-depth cognition of the selected gene, we attempted to perform GO and KEGG pathway enrichment analyses of coexpressed genes. GO analysis showed that CMTM6 coexpressed genes were remarkedly enriched in the BP and CC. The top 10 significant terms of BP and CC are presented in Figures 7A,B. More specifically, coexpressed genes of CMTM6 were enriched in BPs principally involving establishment of protein localization to membrane, protein targeting to membrane, viral gene expression, translational initiation, viral transcription, nuclear-transcribed mRNA catabolic process, nonsense-mediated decay, establishment of protein localization to the endoplasmic reticulum, protein targeting to the ER, cotranslational protein targeting to membrane, SRP-dependent cotranslational protein targeting to membrane, and so on (Figure 7A; Supplementary Table S1). Regarding CCs, the genes were involved in cell–substrate junction, focal adhesion, ribosome, ribosomal subunit, cytosolic ribosome, large and small ribosomal subunit, cytosolic large ribosomal subunit, chromosome, centromeric region, kinetochore, and so on (Figure 7B; Supplementary Table S2). KEGG pathway analysis showed that CMTM6 coexpressed genes were remarkedly enriched in the ribosome, neurotrophin signaling pathway, and pathways in prostate cancer and glioma (Figure 7C; Supplementary Table S3).

### Relationships of CMTM6 Expression With Immune Characteristics

**FIGURE 8:**
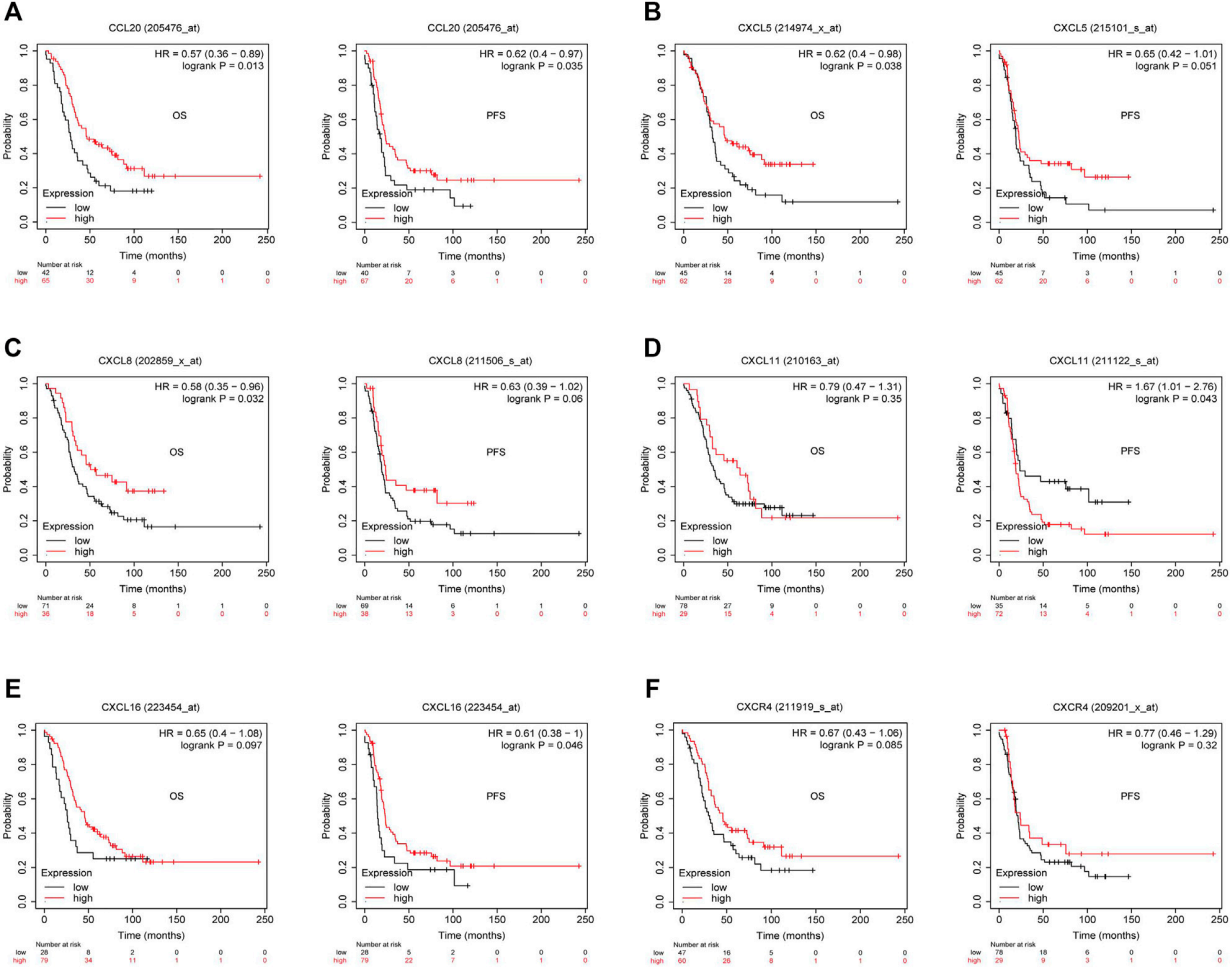
Kaplan–Meier survival curve comparison of the high and low expression of corresponding chemokines and chemokine receptor in GSE26193. **(A–C)** High CCL20, CXCL5, and CXCL8 had better survival in OV. **(D)** Relationship between CXCL11 expression and survival in OV. **(E)** Overexpressed CXCL16 had longer survival in OV. **(F)** Relationship between CXCR4 expression and survival in OV (*p* < 0.05).

Previous studies showed tumor-infiltrating lymphocytes (TILs) as independent predictors of prognosis in cancers (Azimi et al., 2012). Further analysis was performed to probe the immunomodulatory effect of CMTM6. We first detected the correlation of CMTM6 and immune cells in OV using the TIMER, EPIC, and XCELL algorithms of the TIMER2 database. The results suggested associations between tumor purity (*r* = 0.164, *p* = 9.38e-03), CD4^+^ T cell (*r* = 0.263, *p* = 2.64e-05), neutrophils (*r* = 0.199, *p* = 1.60e-03), and myeloid dendritic cells (*r* = 0.162, *p* = 1.05e-02) by CMTM6 expression (Supplementary Figure S3B). Moreover, we performed the association between CMTM6 expression and the immune-related signatures *via* the TISIDB portal, including the abundance of 28 TIL types and immunomodulators (Figures 7A–D). The results exhibited the correlations between CMTM6 and TILs in OV, such as Act_B_ abundance, Act_DC_ abundance, neutrophil_ abundance, Th17_abundance (Figure 7A). Immunomodulators can be further divided into immunoinhibitors, immunostimulators, and MHC molecules. As for immunoinhibitors, CMTM6 expression showed a positive correlation with BTLA_exp and IL10RB_exp and negative correlation with ADORA2A_exp, CD160_exp, KDR_exp, TGFBR1_exp, and TGFB1_exp (Figure 7B). With respect to immunostimulators, CMTM6 expression was positively linked to CD40_exp, HHAL2_exp, IL6_exp, IL6R_exp, KLRC1_exp, TMEM173_exp, and TNFSF13_exp (Figure 7C). Regarding MHC molecules, CMTM6 expression was related to HLA-DMA_exp, HLA-DMB_exp, TAP2_exp, TAPBP_exp, and TNFSF13_exp (Figure 7D). To elucidate the association of CMTM6 with immune cell migration, finally, we comprehensively analyzed the connection with chemokines and chemokine receptors. Figures 7E–J displayed positive correlations of CMTM6 expression with CCL20_exp, CXCL5_exp, CXCL8-exp, CXCL11_exp, CXCL16_exp, and CXCR4_exp. The microarray data sets GSE26913 (*n* = 107) of the Kaplan–Meier plotter disclose that the upregulation of the CCL20, CXCL5, CXCL8, and CXCL16 cohorts had a better prognosis in OV (Figures 8A–C,E), and there was no obvious significance of CXCL11 and CXCR4 expression in the survival of OV (Figures 8D,F). Following that, we searched the prognostic significance of CMTM6 in OV built on GSE26913. Similarly, higher expression of CMTM6 suggests a favorable prognosis (Supplementary Figures S3C,D). Overall, it is seen that CMTM6 possibly played an important role in mediating the OV immune microenvironment and may become a potential biomarker which has crucial impact on prognosis of patients through cross-talk with immune-related factors.

### Relationships Between CMTM6 Expression and Multifarious Immune Markers

To enhance our understanding of CMTM6 cross-talk with immune regulation, we analyzed the relationships between CMTM6 expression and varied immune signatures using the correlation pattern of GEPIA2, including monocytes, TAMs, M1 macrophages, M2 macrophages, B cells, CD8+T cells, T cells (general), NK cells, neutrophils, and dendritic cells. Simultaneously, many T cell subgroups, such as Th1, Th2, Th17, Tfh, Treg, resting Tregs, effector Tregs, effector T cells, naïve T cells, effector memory T cells, resident memory T cells, and exhausted T cells, were analyzed as well (Tables 3, 4). We discovered a prominent positive association of CMTM6 with monocyte markers, natural killer cell markers, neutrophil markers, and dendritic cells markers (Table 3). We also noticed that CMTM6 showed a connection with markers of M1 macrophages and M2 macrophages and appeared to be more correlated with M1 than M2 (Table 3), reminding that CMTM6 is more likely to lead to M1-polarized macrophages in OV, and M1 macrophages have been known to enhance antitumor activity by activating macrophage-mediated inflammation (Ma et al., 2017). In addition to this, CMTM6 was markedly related with 11 of 13 T cell subgroups, and the p-value was all less than 0.01 (Table 4). The information ulteriorly demonstrated that CMTM6 has an influence on immune infiltration cells. Of course, longer-term studies need to be implemented to explore whether CMTM6 is a crucial factor that affects immune infiltration in the tumor microenvironment.

**TABLE 3:** Spearman association between CMTM6 and typical markers of major immune cells in GEPIA2. On the X-axis is CMTM6, and on the Y-axis are cell markers. The bold stands for statistical difference.

Description	Gene markers	R	p-value
Monocyte	CD86/CSF1R/CCR2	0.15	**0.0024**
TAM	CCL2/CD68/IL10	0.11	**0.022**
M1 macrophage	NOS2/IRF5/PTGS2	0.19	**8.6e-05**
M2 macrophage	CD163/VSIG4/MSR1/MRC1	0.12	**0.014**
B cell	CD19/CD79A	−0.092	0.057
CD8^+^ T cell	CD8A/CD8B/CD27	−0.052	0.29
T cell (general)	CD3D/CD3E/CD2	0.0034	0.94
Natural killer cell	FCGR3A/NCAM1/CD94/KIR2DL3/CD161	0.17	**3e-04**
Neutrophils	ITGAM/CEACAM8/CCR7	0.18	**0.00013**
Dendritic cell	HLA-DPB1/HLA-DQB1/HLA-DRA/HLA-DPA1/CD1C/NRP1/ITGAX	0.12	**0.012**

**TABLE 4:** Spearman connection between CMTM6 and representative markers of different T cell subsets in GEPIA2. On the X-axis is CMTM6, and on the Y-axis are cell markers. The bold stands for statistical difference.

Description	Gene markers	R	p-value
Th1	TBX21/STAT4/STAT1/IFNG/TNF	0.21	**8.9e−06**
Th2	GATA3/STAT6/STAT5A/IL13	0.27	**2.1e−08**
Th17	STAT3/IL17A	0.3	**1.5e−10**
Tfh	BCL16/IL21	0.3	**1.6e−10**
Treg	FOXP3/CCR8/STAT5B	0.22	**4.4e−06**
Effector treg T-cell	FOXP3/CCR8/TNFRSF9	0.19	**5.1e−05**
Resting treg	FOXP3/IL2RA	0.17	**0.00051**
Effector T-cell	CX3CR1/FGFBP2/FCGR3A	0.16	**0.0012**
Naïve T-cell	CCR7/SELL	0.13	**0.0053**
Effector memory T-cell	DUSP4/GZMK/GZMA	0.085	0.081
Resident memory T-cell	CD69/CXCR6/MYADM	0.18	**0.00014**
General memory T-cell	CCR7/SELL/IL7R	0.17	**0.00053**
Exhausted T-cell	PDCD1/HAVCR2/TIGIT/CXCL13/LAYN	0.049	0.32

### Copy Number Variation, Methylation, and Mutation Analysis of CMTM6

**FIGURE 9:**
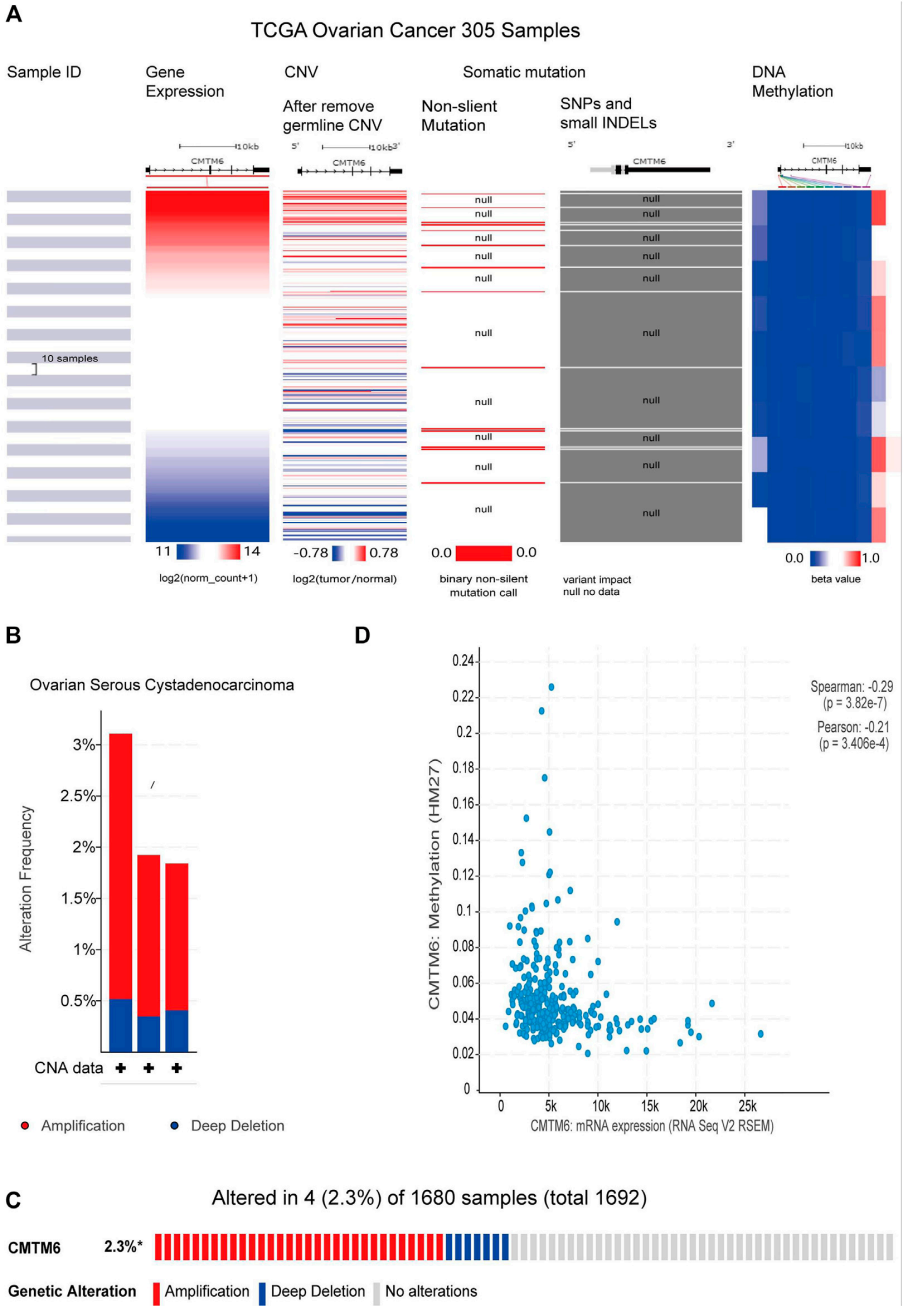
CNV, methylation, and mutation analysis of CMTM6 in OV (UCSC Xena and cBioPortal). **(A)** Heatmap viewing the associations between CMTM6 mRNA and CNV, methylation, and somatic mutations in OV. **(B,C)** Summary of genetic alteration feature of CMTM6 within OV. CMTM6 was altered in four samples of 1,680 samples with OV (2.3%). **(D)** Relation of CMTM6 expression and the methylation level for OV.

CNV refers to the insertion, deletion, duplication, translocation, and derived chromosomal structural variation of DNA fragments, which has been a pivotal factor in genetic variation and biodiversity (Iafrate et al., 2004). DNA methylation is a vital mode of regulation of gene expression, and hyper-methylation of promoter regions reduces gene expression (Saghafinia et al., 2018). Previous gene resequencing efforts have acknowledged that gene mutation is associated with human cancers (Citron et al., 2002). Because of CMTM6 expression being obviously increased in OV than control tissues, we tried to analyze the links between CMTM6 level with CNV, DNA methylation, and gene mutation by the UCSC Xena database. The heatmap revealed that CMTM6 expression was related with CNV and DNA methylation, but not with somatic mutation in OV (Figure 9A). Next, we analyzed the genetic alterations of CMTM6 in the TCGA-OV cohorts through the cBioPortal online tool. Two kinds of alterations (amplification and deletion) were detected in TCGA sources of OV (Figure 9B). CMTM6 was altered in four samples of 1,680 samples with OV, occupying 2.3% (Figure 9C). Interestingly, there was no relationship between the genetic alteration of CMTM6 and clinical prognosis of OV (Supplementary Figures S3E,F). After that, the DNA methylation status of CMTM6 was analyzed; there was a negative link between CMTM6 expression and the methylation level for OV (Figure 9D *p* = 3.28e-7, *r* < 0, Spearman’s analysis; *p* = 3.406e-4, *r* < 0, Pearson’s analysis). Considering all this, CNV and DNA methylation might contribute to the elevated level of CMTM6 in OV.

## Discussion

Among gynecological oncology, ovarian cancer leads the way in death rates (Konstantinopoulos et al., 2020). OV is a tumor with a unique set of clinicopathological and molecular features and prognosis. Despite there being a variety of OV subtypes, these are viewed as a single disease (Matsuo et al., 2020). Extensive work has been performed to characterize these subtypes and to identify pathways and potential biomarkers associated with OV (Gentry-Maharaj et al., 2020). The identification of neoteric biomarkers for OV is decisive to its diagnosis, therapy, and prognosis. So, our pressing need is to excavate a new associated biomarker to predict patient prognosis and ensure the probable molecular mechanisms behind the response to therapy.

CMTM6 is a widely expressed protein that plays a central role in membrane protein transport, transmembrane, and secreted protein (Delic et al., 2015). So far, dysregulation of the CMTM6 gene has been reported in many cancers, including head and neck squamous cell carcinoma (Chen et al., 2020), metastatic melanoma (Martinez-Morilla et al., 2020), non–small cell lung cancer (Zugazagoitia et al., 2019), and triple-negative breast cancer (Tian et al., 2021). Curiously, CMTM6 expression’s association with OV is not clear. We believe this is the first study to explore the correlation of CMTM6 with prognosis of OV. This study helps increase awareness of treatment options and with an augment of the accuracy of prognosis for patients with OV.

We first identified high expression of CMTM6 in OV samples and cell lines as compared with normal samples and IOSE80 cell line, respectively. There is no apparent relevance between CMTM6 expression and grouped in advanced cancer stages and tumor grade nor with patient’s race, age, and TP53-mutation status. Of course, more clinical samples are needed to verify or overturn this phenomenon. It is worth special attention that the most clinically relevant finding was that high expression of CMTM6 was associated with good survival in data of TCGA. Furthermore, Kaplan–Meier plotter and PrognoScan database analyses also demonstrated that CMTM6 overexpression in OV patients had prolonged survival than those with low expression. The abovementioned observation strongly substantiates that CMTM6 is likely to be a latent prognostic marker in OV.

GSEA analysis revealed the function of CMTM6 enriched in immune response–related pathways, such as natural killer cell–mediated cytotoxicity, chemokine signaling pathway, B cell receptor signaling pathway, and T cell receptor signaling pathway. NK cells, as antitumor immune cells, play an indispensable role in killing tumor cells and exerting immune function (Uchida and Moore, 1984). Functional analysis also exhibited that CMTM6 was linked to multiple metabolism pathways. Among these metabolism pathways, it was reported that ubiquitin-mediated proteolysis could serve as a tumor suppressant in OV (Ji et al., 2021) . These may have an effort on the longer survival time in the group with high CMTM6 expression. Most previous studies have shown the tumorigenicity of CMTM6 in the development and progression of cancer (Guan et al., 2018; Chen et al., 2020; Zheng et al., 2020); there are few studies that discussed the tumor-suppressive effect of CMTM6 (Mamessier et al., 2018). Until now, the molecular mechanism by which CMTM6 acts as a tumor suppressor factor has been poorly understood. Our research provides further evidence that CMTM6 has dual effects in the formation of different solid tumors, which may be the reason why CMTM6 regulates different mechanisms. The PPI network was formed by inputting the first 100 genes coexpressed by CMTM6 into the GeneMANIA database to clarify their interactions. Functional enrichment analysis followed to characterize genes coexpressed with CMTM6. The GO enrichment analysis manifested that these genes were distinctly involved in establishment of protein localization to membrane and cell–substrate junction. Also, KEGG pathway enrichment analysis manifested that these genes were eminent in the ribosome.

Tumor cells exist in a complex tumor environment (TME) (Tower et al., 2019). The essence of the TME is the cellular and non-cellular components present in and around the tumor. Generally, the TME is subdivided into the extracellular matrix (ECM), stromal cells, and immune cells (Yong et al., 2019). The ability to predict and guide immunotherapy response has great prospects as researchers gain a better understanding of the tumor immune microenvironment (Bedognetti et al., 2016). TIMER2.0 and TISIDB databases revealed the positive association between CMTM6 expression and interrelated TILs, immunoinhibitors, immunostimulators, MHC molecules, chemokines, and chemokine receptors. Following that, analysis of CMTM6 and immune infiltration cell marker genes implicated that CMTM6 was also positively correlated with the marker genes of T helper cells (Th1, Th2, Tfh, Th17, and Tregs) and macrophages (M1 and M2). Th1, Tfh, Th17, and M1 in OV also forecasted favorable prognosis (Dobrzanski et al., 2009; Crotty, 2019; Travers et al., 2019; Block et al., 2020), and the study suggested that CMTM6 might recruit and regulate T cell and macrophage function to affect tumor regression in OV.

Epigenetic phenomena play an essential role in regulating genetic expression (Grewal and Rice, 2004). As a result, we found that CMTM6 expression was closely related to CNV and DNA methylation, but not with somatic mutations. Further genetic analysis indicated that genetic alteration (amplification and deletion) frequency of CMTM6 of OV was only 2.3% and was without relationship with prognosis, which greatly reduces the likelihood of high expression of OV. In addition, we detected that CMTM6 gene expression was strongly negatively associated with methylation level, which is usually equivalent to that of transcription inhibitors (Wang et al., 2013).

Our present study is not without limitations. Most notably, the majority of our ratiocinations were based on online integrative bioinformatics analysis tools. Hence, it is imperative to obtain more reliable data derived from *in vitro* or *in vivo* experiments, as well as clinical validation, to explore the specific regulatory mechanisms between CMTM6 expression and genetic alterations, and momentous pathways selected by GSEA analysis in OV are imperative. In addition to this, to ensure greater reliability and representativeness of the results and assumptions, the samples should be expanded for further research. In the future, we intend to manipulate deep investigations in the role of CMTM6 in the occurrence and development of OV to support our conclusion that CMTM6 could be considered an available prognostic biomarker. Fortunately, a collection of plans has been formulated for some recent laboratory work.

## Conclusion

Taken together, this study disclosed for the first time that increased CMTM6 expression in OV was an independent prognostic biomarker that prolonged survival, where immune-related and metabolic pathways favored this behavior. We hope that our findings will aid future research and help clinicians in the choice of appropriate measures for their patients and improve the long-term outcome of OV.

## Data Availability

The datasets presented in this study can be found in online repositories. The names of the repository/repositories and accession number(s) can be found in the article/Supplementary Material.
