# Supplementary material for: Overexpressed CMTM6 Improves Prognosis and Associated With Immune Infiltrates of Ovarian Cancer
Source: Front Mol Biosci. 2022 Jan 31;9:769032. doi: 10.3389/fmolb.2022.769032 (PMC8841687; doi:10.3389/fmolb.2022.769032)
Supplement: Supplementary file 1 [file DataSheet1.DOCX]

Supplementary Material

# Supplementary Figures and Tables

## Supplementary Figures

**
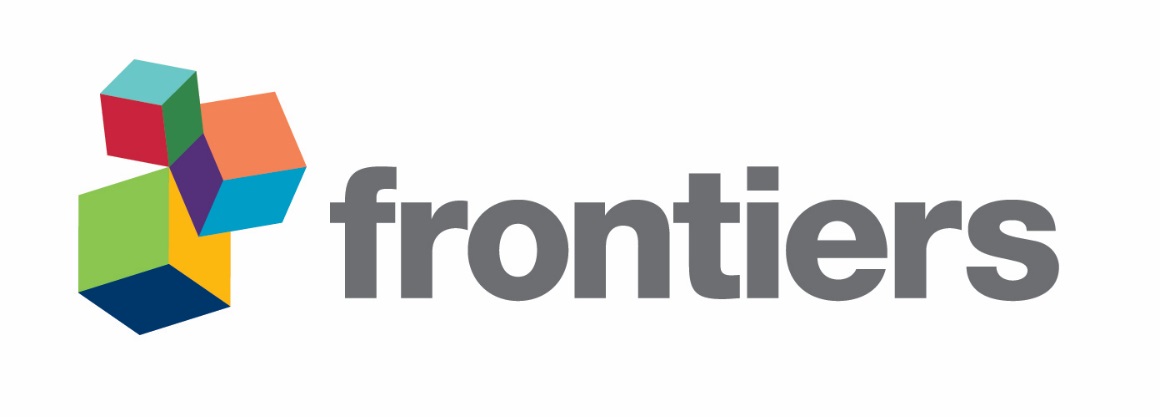
**

**Supplementary Figure 1.** CMTM6 expression in OV based on different clinical parameters from the UALCAN, including (A) individual cancer stages, (B) patient 's race, (C) patient 's age, (D) tumor grade, (E) TP53 mutation status.


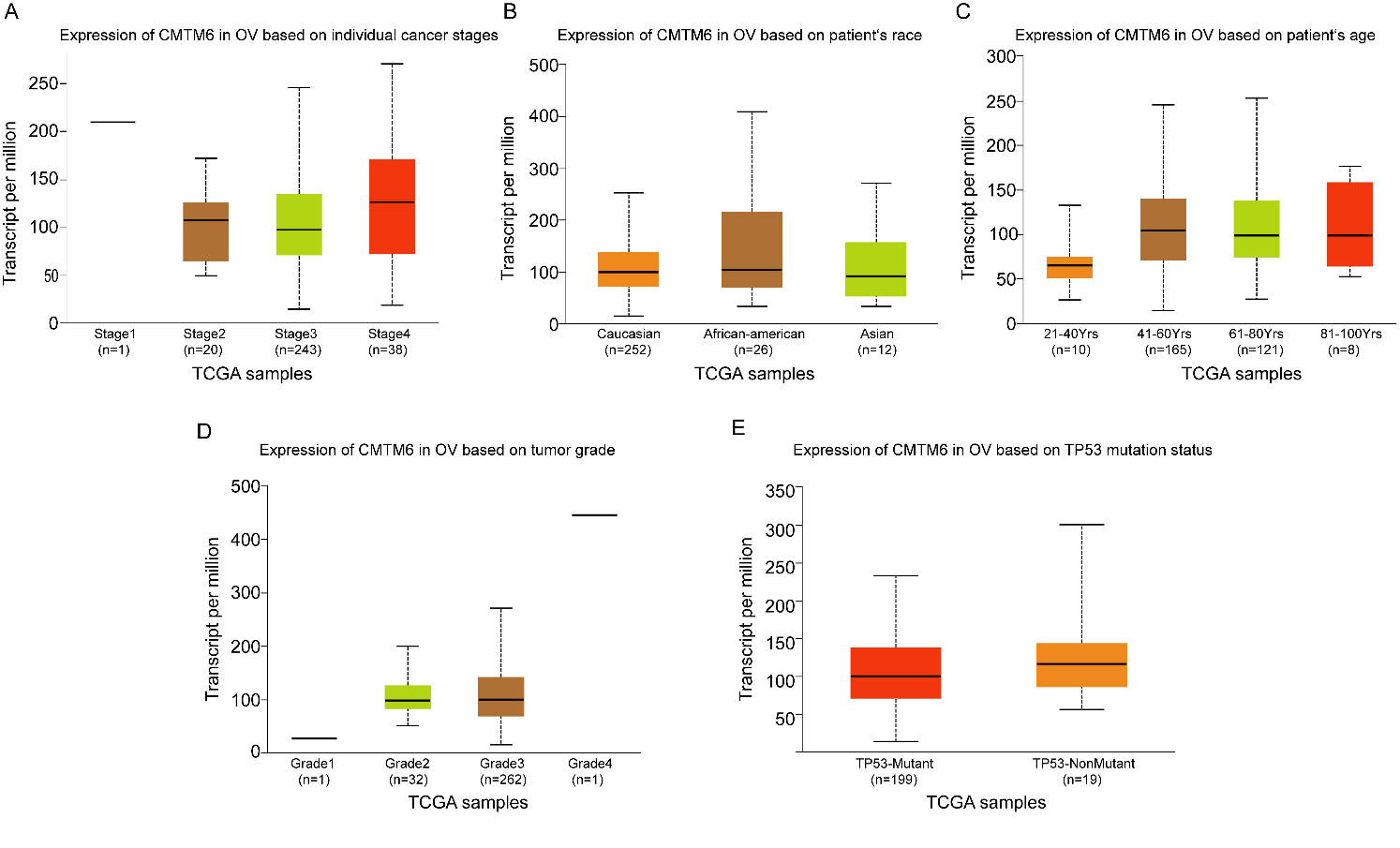


**Supplementary Figure 2.** Survival analysis of CMTM6 expression in (A) LGG (A), PAAD (B), KIRC (C), and LIHC (D) (OS in GEPIA2 and Kaplan-Meier plotter). P < 0.05 represents statistical significance (LGG, Brain Lower Grade Glioma; PAAD, Pancreatic adenocarcinoma; KIRC, Kidney renal clear cell carcinoma; LIHC, Liver hepatocellular carcinoma).


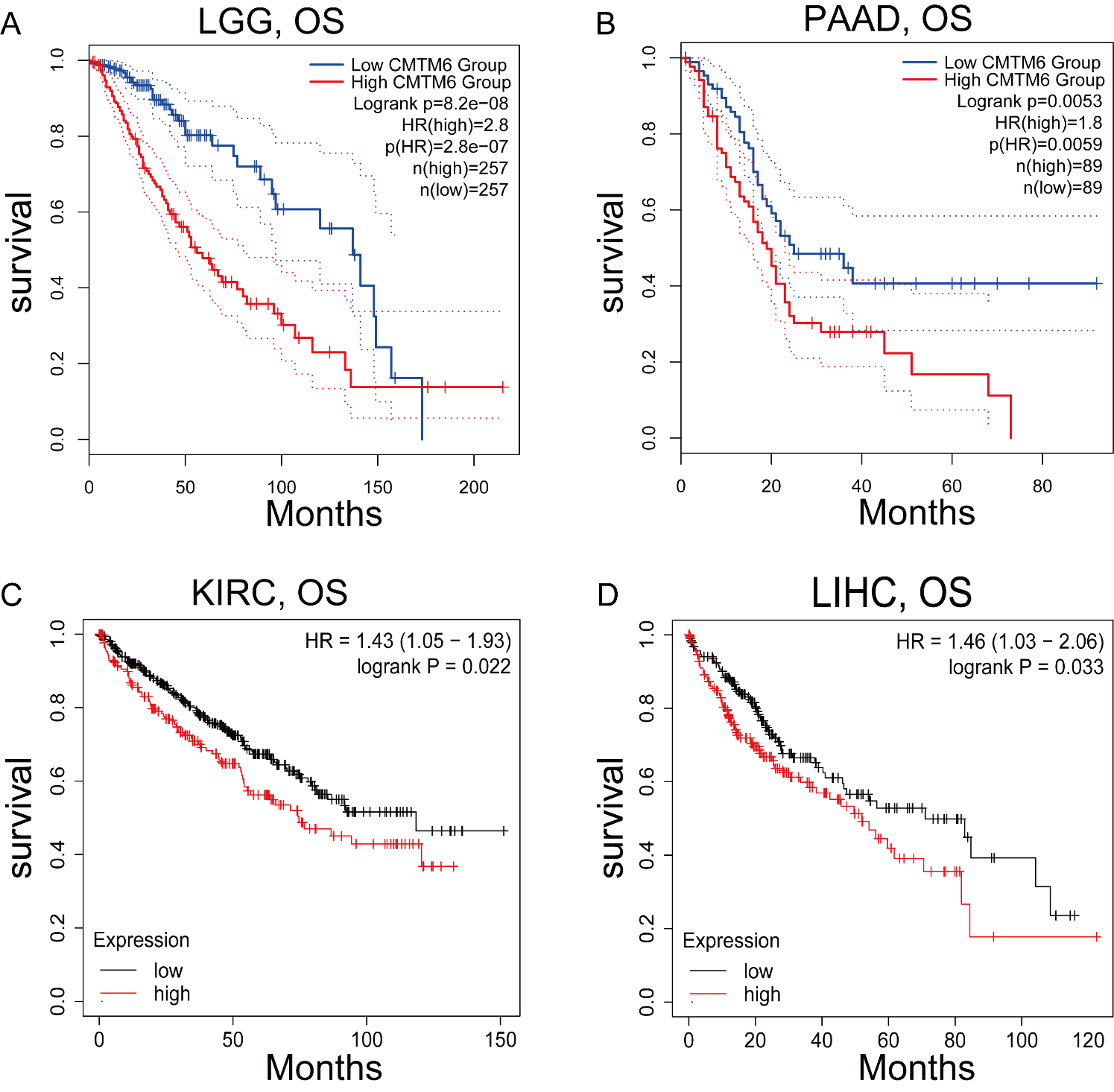


**Supplementary Figure 3.** (A) GSEA analysis of some metabolism pathways of CMTM6 for OV; (B) Correlations between CMTM6 expression with tumor immune infiltration levels of CD8+ T cells, CD4+ T cells, B cells, macrophage cells, neutrophil cells, and myeloid dendritic cells by TIMER2.0 database; (C, D) Survival analysis of CMTM6 expression in GSE26913; (E, F) The correlation between the genetic alteration of *CMTM6* and the OS and PFS prognosis of OV cases.


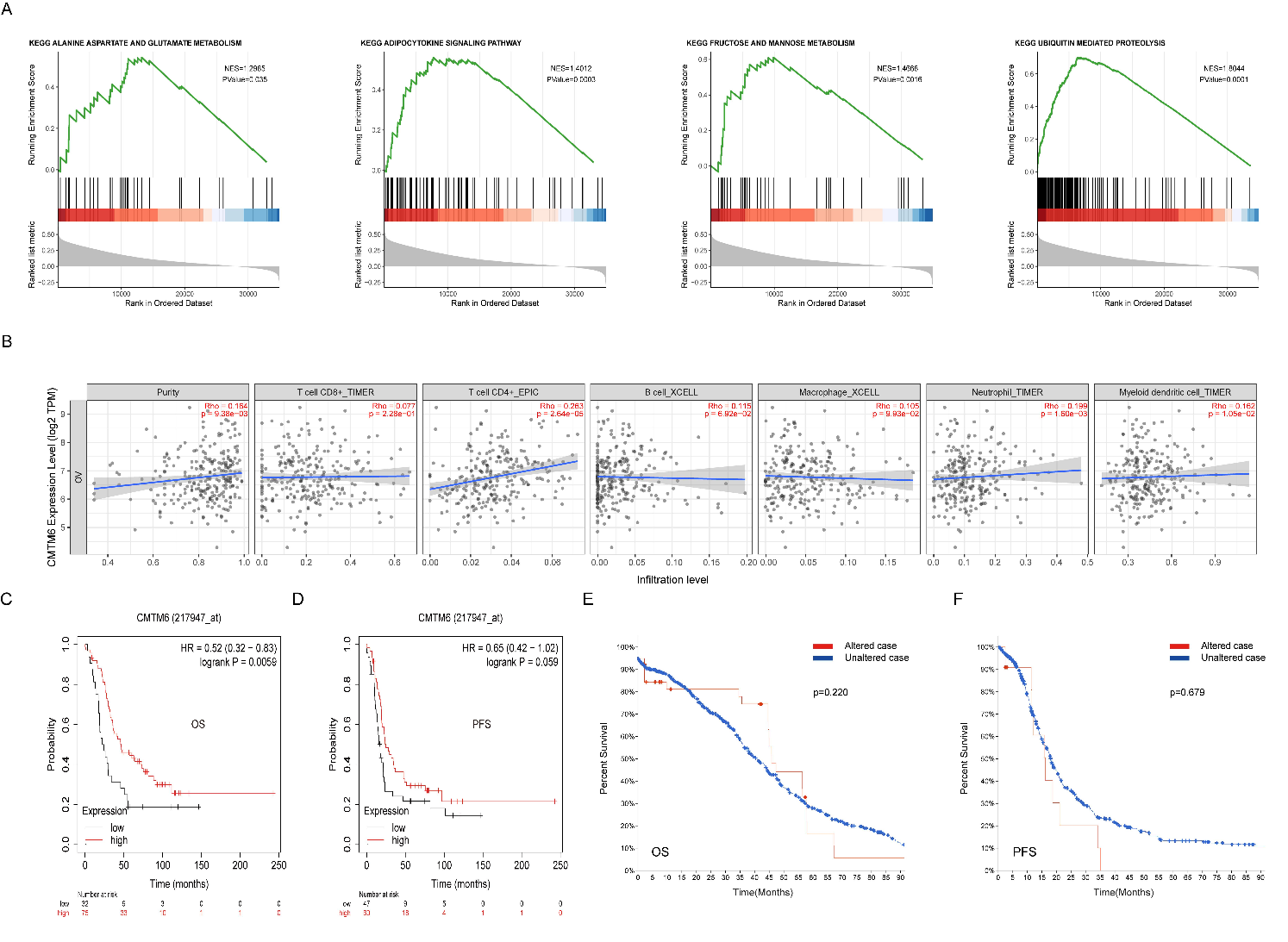


# Supplementary Table 1. BP part of GO terms from enrichment analysis of co-expressed genes with CMTM6 in OV

| ID | Term | P.adjust | Counts | Gene names |
| --- | --- | --- | --- | --- |
| GO:0000184 | nuclear-transcribed mRNA catabolic process, nonsense-mediated decay | 2.36e-07 | 10 | RPL14/RPL37A/RPL38/RPS11/RPL23A/RPS19/RPLP2/RPL27A/RPS10/NBAS |
| GO:0006614 | SRP-dependent cotranslational protein targeting to membrane | 8.16e-07 | 9 | RPL14/RPL37A/RPL38/RPS11/RPL23A/RPS19/RPLP2/RPL27A/RPS10 |
| GO:0006613 | cotranslational protein targeting to membrane | 8.26e-07 | 9 | RPL14/RPL37A/RPL38/RPS11/RPL23A/RPS19/RPLP2/RPL27A/RPS10 |
| GO:0006612 | protein targeting to membrane | 1.02e-06 | 11 | AKT2/RPL14/RPL37A/RPL38/RPS11/RPL23A/RPS19/RPLP2/RPL27A/RPS10/ZDHHC20 |
| GO:0045047 | protein targeting to ER | 1.08e-06 | 9 | RPL14/RPL37A/RPL38/RPS11/RPL23A/RPS19/RPLP2/RPL27A/RPS10 |
| GO:0072599 | establishment of protein localization to endoplasmic reticulum | 1.20e-06 | 9 | RPL14/RPL37A/RPL38/RPS11/RPL23A/RPS19/RPLP2/RPL27A/RPS10 |
| GO:0090150 | establishment of protein localization to membrane | 1.29e-06 | 13 | AKT2/RPL14/RPL37A/RPL38/RPS11/RPL23A/RPS19/RPLP2/RPL27A/RPS10/CALM3/CNST/ZDHHC20 |
| GO:0019083 | viral transcription | 1.57e-06 | 10 | RPL14/RPL37A/RPL38/RPS11/RPL23A/RPS19/RPLP2/RPL27A/RPS10/TPR |
| GO:0006413 | translational initiation | 2.72e-06 | 10 | RPL14/RPL37A/RPL38/RPS11/RPL23A/RPS19/RPLP2/RPL27A/RPS10/TPR |
| GO:0019080 | viral gene expression | 3.12e-06 | 10 | RPL14/RPL37A/RPL38/RPS11/RPL23A/RPS19/RPLP2/RPL27A/RPS10/TPR |
| GO:0070972 | protein localization to endoplasmic reticulum | 3.92e-06 | 9 | RPL14/RPL37A/RPL38/RPS11/RPL23A/RPS19/RPLP2/RPL27A/RPS10/TPR |
| GO:0000956 | nuclear-transcribed mRNA catabolic process | 4.16e-06 | 10 | RPL14/RPL37A/RPL38/RPS11/RPL23A/RPS19/RPLP2/RPL27A/RPS10/NBAS |
| GO:1903035 | negative regulation of response to wounding | 0.000416 | 6 | PRKG1/CASK/SPP1/PDGFA/CLASP1/PHLDB2 |
| GO:0006605 | protein targeting | 0.000494 | 11 | AKT2/RPL14/RPL37A/RPL38/RPS11/RPL23A/RPS19/RPLP2/RPL27A/RPS10/ZDHHC20 |
| GO:0006402 | mRNA catabolic process | 0.000737 | 10 | RPL14/RPL37A/RPL38/RPS11/RPL23A/RPS19/RPLP2/RPL27A/RPS10/NBAS |
| GO:0006401 | RNA catabolic process | 0.00164 | 10 | RPL14/RPL37A/RPL38/RPS11/RPL23A/RPS19/RPLP2/RPL27A/RPS10/NBAS |
| GO:0061045 | negative regulation of wound healing | 0.00211 | 5 | PRKG1/CASK/PDGFA/CLASP1/PHLDB2 |
| GO:0008286 | insulin receptor signaling pathway | 0.00428 | 6 | AKT2/ENPP1/ATP6V0E1/APC/PIK3C2A/PTPN11 |
| GO:0042254 | ribosome biogenesis | 0.00625 | 8 | WDR36/FCF1/SDAD1/RPL14/RPL38/RPL23A/RPS19/RPS10 |
| GO:1903034 | regulation of response to wounding | 0.00826 | 6 | PRKG1/CASK/SPP1/PDGFA/CLASP1/PHLDB2 |
| GO:0000028 | ribosomal small subunit assembly | 0.00948 | 3 | RPL38/RPS19/RPS10 |
| GO:0042255 | ribosome assembly | 0.0159 | 4 | RPL38/RPL23A/RPS19/RPS10 |
| GO:0061041 | regulation of wound healing | 0.0230 | 5 | PRKG1/CASK/PDGFA/CLASP1/PHLDB2 |
| GO:0042273 | ribosomal large subunit biogenesis | 0.0230 | 4 | SDAD1/RPL14/RPL38/RPL23A |
| GO:0051497 | negative regulation of stress fiber assembly | 0.0261 | 3 | TMEFF2/CLASP1/PHLDB2 |
| GO:0032232 | negative regulation of actin filament bundle assembly | 0.0307 | 3 | TMEFF2/CLASP1/PHLDB2 |
| GO:0044319 | wound healing, spreading of cells | 0.0307 | 3 | TMEFF2/CLASP1/PHLDB2 |
| GO:0090505 | epiboly involved in wound healing | 0.0307 | 3 | TMEFF2/CLASP1/PHLDB2 |
| GO:0032869 | cellular response to insulin stimulus | 0.0313 | 6 | AKT2/ENPP1/ATP6V0E1/APC/PIK3C2A/PTPN11 |
| GO:0090504 | epiboly | 0.0313 | 3 | TMEFF2/CLASP1/PHLDB2 |
| GO:0071375 | cellular response to peptide hormone stimulus | 0.0356 | 7 | CDC5L/AKT2/ENPP1/ATP6V0E1/APC/PIK3C2A/PTPN11 |
| GO:1901203 | positive regulation of extracellular matrix assembly | 0.0492 | 2 | CLASP1/PHLDB2 |
| GO:2000659 | regulation of interleukin-1-mediated signaling pathway | 0.0492 | 2 | ZNF675/OTUD4 |

# Supplementary Table 2. CC part of GO terms from enrichment analysis of co-expressed genes with CMTM6 in OV.

| ID | Term | P.adjust | Counts | Gene names |
| --- | --- | --- | --- | --- |
| GO:0022626 | cytosolic ribosome | 3.11e-07 | 9 | RPL14/RPL37A/RPL38/RPS11/RPL23A/RPS19/RPLP2/RPL27A/RPS10 |
| GO:0044391 | ribosomal subunit | 1.49e-06 | 10 | RPL14/RPL37A/RPL38/RPS11/RPL23A/RPS19/RPLP2/RPL27A/RPS10/MRPS15 |
| GO:0005925 | focal adhesion | 3.27e-06 | 13 | TPM4/NCKAP1/HSP90B1/AHNAK/CASK/RPL37A/RPL38/RPS11/RPS19/RPLP2/RPS10/CLASP1/PHLDB2 |
| GO:0030055 | cell-substrate junction | 3.27e-06 | 13 | TPM4/NCKAP1/HSP90B1/AHNAK/CASK/RPL37A/RPL38/RPS11/RPS19/RPLP2/RPS10/CLASP1/PHLDB2 |
| GO:0005840 | ribosome | 7.05e-06 | 10 | RPL14/RPL37A/RPL38/RPS11/RPL23A/RPS19/RPLP2/RPL27A/RPS10/MRPS15 |
| GO:0022625 | cytosolic large ribosomal subunit | 8.74e-06 | 6 | RPL14/RPL37A/RPL38/RPL23A/RPLP2/RPL27A |
| GO:0015934 | large ribosomal subunit | 0.000471 | 6 | RPL14/RPL37A/RPL38/RPL23A/RPLP2/RPL27A |
| GO:0015935 | small ribosomal subunit | 0.0106 | 4 | RPS11/RPS19/RPS10/MRPS15 |
| GO:0000776 | kinetochore | 0.0106 | 5 | NUDCD2/APC/CLASP1/CBX5/TPR |
| GO:0022627 | cytosolic small ribosomal subunit | 0.0287 | 3 | RPS11/RPS19/RPS10 |
| GO:0005765 | lysosomal membrane | 0.0328 | 7 | CMTM6/AHNAK/ENPP1/RHEB/B4GALT1/CD74/ATP11B |
| GO:0098852 | lytic vacuole membrane | 0.0328 | 7 | CMTM6/AHNAK/ENPP1/RHEB/B4GALT1/CD74/ATP11B |
| GO:0000775 | chromosome, centromeric region | 0.0366 | 5 | NUDCD2/APC/CLASP1/CBX5/TPR |
| GO:0035577 | azurophil granule membrane | 0.0401 | 3 | CMTM6/B4GALT1/ATP11B |
| GO:0005774 | vacuolar membrane | 0.0475 | 7 | CMTM6/AHNAK/ENPP1/RHEB/B4GALT1/CD74/ATP11B |

**Supplementary Table 3.** KEGG terms from enrichment analysis of co-expressed genes with CMTM6 in OV

| ID | Term | P value | Counts | Gene names |
| --- | --- | --- | --- | --- |
| hsa03010 | Ribosome | 1.08e-08 | 9 | RPL14/RPL37A/RPL38/RPS11/RPL23A/RPS19/RPLP2/RPL27A/RPS10 |
| hsa04722 | Neurotrophin signaling pathway | 0.0122 | 4 | APC/AKT2/CALM3/PTPN11 |
| hsa05215 | Prostate cancer | 0.0250 | 3 | HSP90B1/AKT2/PDGFA |
| hsa05214 | Glioma | 0.0108 | 3 | AKT2/CALM3/PDGFA |
